# Supplementary material for: Aneurysm-on-a-Chip: Setting Flow Parameters for Microfluidic Endothelial Cultures Based on Computational Fluid Dynamics Modeling of Intracranial Aneurysms
Source: Brain Sci. 2022 May 5;12(5):603. doi: 10.3390/brainsci12050603 (PMC9139202; doi:10.3390/brainsci12050603)
Supplement: Supplementary file 1 [file brainsci-12-00603-s001.zip › Supplementary file S2 - Materials for the microfluidic setup.pdf]

Supplementary file 2 - Materials for the microfluidic setup

Blunt needles 18G, ½" (Cat#: 918050-TE, Farnell)

FEP Tubing 1/16" OD x .020" ID Natural 50ft. Cat#: 1548L (IDEX, USA)

Cole-Parmer Masterflex Transfer Tubing, Tygon ND-100-80 Microbore, 0.020" ID x 0.060" OD; 100 ft (Cole-Parmer, USA).

Translucent Silicone Tubing (ID 1 mm, OD 3 mm; Versitec, Canada)

Fluigent L-Switch and Switch board with respective peek connectors and ethernet cables.

P-cap for 15ml Low pressure package (Fluigent, France)

Flow sensor – flow unit L (FLU-L-D, Fluigent, France)

Flowboard, flow sensor hub to connect the flow sensors. (Fluigent, France)

Pressure controller MFCS-EX with integrated compressor and 8 regulated channels (0 to 345 mbar channel part number EX-00345001) (Fluigent, France)

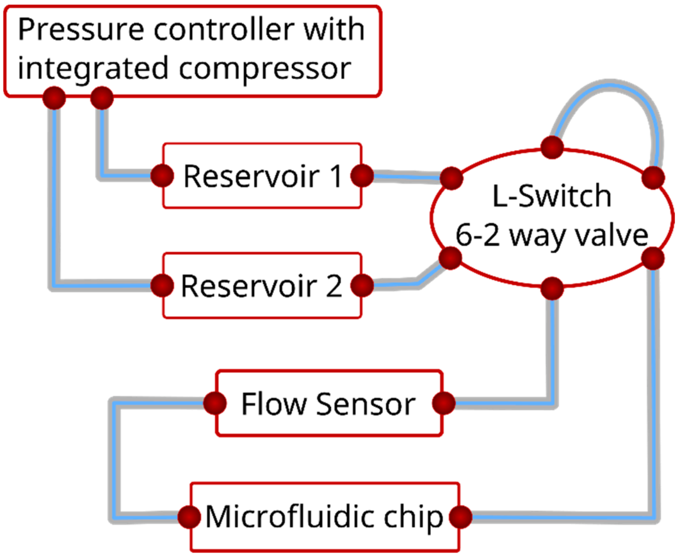

Figure S2. Schematic view of the fluidic circuit and the respective connection among all the different components.
